# Protocol for a mixed-methods study of supplemental oxygen in pulmonary fibrosis

**DOI:** 10.1186/1471-2466-14-169

**Published:** 2014-11-01

**Authors:** Amanda Belkin, Kaitlin Fier, Karen Albright, Susan Baird, Brenda Crowe, Linda Eres, Marjorie Korn, Leslie Maginn, Mark McCormick, Elisabeth D Root, Thomas Vierzba, Frederick S Wamboldt, Jeffrey J Swigris

**Affiliations:** Autoimmune Lung Center and Interstitial Lung Disease Program, National Jewish Health, Southside Building, Office #G011 1400 Jackson Street, Denver, CO 80206 USA; Department of Community and Behavioral Health, Colorado School of Public Health, and Colorado Health Outcomes Program, University of Colorado School of Medicine, Aurora, CO USA; Participation Program for Pulmonary Fibrosis (P3F), Denver, CO USA; Exempla Lutheran Medical Center, Wheat Ridge, CO USA; Department of Geography, University of Colorado, Boulder, CO USA; Division of Pulmonary, Critical Care and Sleep Medicine, Sleep and Behavioral Health Sciences Section, National Jewish Health, Denver, CO USA

## Abstract

**Background:**

Little is known about whether or how supplemental oxygen affects patients with pulmonary fibrosis.

**Methods/Design:**

A mixed-methods study is described. Patients with pulmonary fibrosis, informal caregivers of pulmonary fibrosis patients and practitioners who prescribe supplemental oxygen will be interviewed to gather data on perceptions of how supplemental oxygen impacts patients. In addition, three hundred pulmonary fibrosis patients who do not use daytime supplemental oxygen will be recruited to participate in a longitudinal, pre-/post- study in which patient-reported outcome (PRO) and activity data will be collected at baseline, immediately before daytime supplemental oxygen is initiated, and then once and again 9–12 months later. Activity data will be collected using accelerometers and portable GPS data recorders. The primary outcome is change in dyspnea from before to one month after supplemental oxygen is initiated. Secondary outcomes include scores from PROs to assess cough, fatigue and quality of life as well as the activity data. In exploratory analyses, we will use longitudinal data analytic techniques to assess the trajectories of outcomes over time while controlling for potentially influential variables.

**Discussion:**

Throughout the study and at its completion, results will be posted on the website for our research program (the Participation Program for Pulmonary Fibrosis or P_3_F) at http://www.pulmonaryfibrosisresearch.org.

## Background

Pulmonary fibrosis (PF) is a chronic lung disease in which the normally thin, delicate alveolar walls are infiltrated by extracellular matrix and mature, inelastic collagen, leaving them irreversibly thickened and dysfunctional [1]. The stiff lungs of patients with PF hold less air than normal and do not allow transfer of oxygen from airspace to bloodstream. Clinically, this leads to low blood oxygen levels and shortness of breath.

PF can be caused by a number of entities, including connective tissue diseases (e.g., rheumatoid arthritis), but most commonly, it exists as an idiopathic condition, in which case, despite extensive investigation, its cause remains unknown. Whether of known-cause or idiopathic, PF is a challenging disease for patients to live with: it typically progresses and shortens survival; its symptoms—shortness of breath, nagging cough and fatigue—limit physical and social activities and impair patients’ quality of life (QOL) [2, 3]; and there is no cure.

At some point in their illness, many PF patients will develop low blood oxygen levels when sleeping, exerting and/or at rest [4, 5]. Such patients may be prescribed supplemental oxygen (O_2_) to maintain normal blood oxygen levels. Current research suggests PF patients and their informal caregivers (spouse/partner/loved one) typically view O_2_ with extreme resignation: it is a visible reminder of disease progression; the equipment can be heavy and cumbersome to lug around; and having to deal with O_2_ forces patients to strategize before leaving home for the day (extended travel creates even greater logistical issues). In short, O_2_ is viewed by many PF patients as an unwanted but necessary burden [6, 7].

Studies have shown that, in certain patients with chronic obstructive pulmonary disease (COPD), O_2_ can prolong survival, improve physical functioning and improve QOL [8, 9]. But even the data for O_2_ in COPD—one of the most common respiratory diseases in the world—are surprisingly scant. And despite the nearly universal need for O_2_ among PF patients, even less is known about whether or how O_2_ affects their physical functioning, symptoms and QOL [10, 11]. Currently, because of the paucity of data to inform them, PF patients and prescribers lack sound, quantifiable evidence needed to answer patients when they ask critical questions, such as, why is supplemental oxygen important? And what can I expect to gain by using oxygen?

The members of our team—called the Participation Program for Pulmonary Fibrosis (P_3_F)—which includes professionals as well as people living with PF, have designed a rigorous mixed-methods study in an attempt to systematically answer these and other questions about O_2_ in PF (see Table 1). Subjects recruited from across the nation will have qualitative, quantitative or both types of data collected either once or up to four times, depending on whether they use O_2_ during the day at enrollment. In the qualitative piece, we are collecting perceptions of O_2_ from PF patients, informal caregivers (ICs) of PF patients and from prescribers of O_2_ to PF patients. In the quantitative piece, we are using a pre-/post- study design to measure the effect of O_2_ on a wide range of patient-centered outcomes, including dyspnea, QOL, fatigue, cough, day-to-day functioning and activity space (a medical geography term referring to the extent of the environment used by an individual) in patients with PF.

**Table 1:** **Mixed-methodology study design**

Category	N	Study design	Method	Data collection
1. PF patients who have used O_2_ for at least eight months	20	X-sectional	QUAL	Interview
			QUANT	PRO Questionnaires
2. ICs of PF patients who have used O_2_ for at least eight months	20	X-sectional	QUAL	Interview
3. Practitioners who have prescribed O_2_ for at least one patient with PF	20	X-sectional	QUAL	Interview
4. PF patients not on daily-use O_2_ at enrollment	40	Longitudinal	QUAL	Interviews
			QUANT	PRO Questionnaires
				GPS
				Accelerometer
	260	Longitudinal	QUANT	PRO Questionnaires
				GPS
				Accelerometer

QUAL = qualitative data collection; QUANT = quantitative data collection; PRO = patient-reported outcome measure; GPS = global positioning system.

We will test the working hypothesis that compared with just prior to starting O_2_, after one month of use, outcomes will be better (primary outcome is shortness of breath with secondary outcomes being QOL, fatigue, cough, functioning and activity space). A secondary hypothesis we will test is compared with just prior to starting O_2_, these outcomes will remain improved after 9–12 months. In this manuscript, we detail the study design.

## Methods

### Enrollment

Study participants will be recruited through an assortment of efforts. Our main method of advertising is through the P_3_F website (http://www.pulmonaryfibrosisresearch.org) where people can find information about the study and contact P_3_F coordinators, or they have the option to sign up for our Contact Registry which gives P_3_F coordinators permission to contact patients directly. We have also enlisted the help of patients and professionals from all over the US—physicians, nurses, research coordinators, support group leaders—to spread the word about our program and study. We have also utilized other internet-based outlets such as online support groups and social media (Facebook and Twitter) to increase the reach of our program and study.

We will enroll subjects into one of four categories: 1) PF patients who use daytime O_2_; 2) ICs of PF patients who use daytime O_2_; 3) physicians who prescribe daytime O_2_ to PF patients; and 4) PF patients who do not use daytime O_2_ (they will participate in the pre-/post- O_2_ longitudinal study). The consent process will be conducted via mail, e-mail or fax for all patient-participants and over the phone for ICs and physicians.

### Inclusion criteria

All persons enrolled in the study must be able to speak and read English and be at least 18 years of age. Patients will be eligible to participate if they have a diagnosis of PF and, at time of enrollment, have either been using daytime oxygen for at least eight months or are not using daytime O_2_ at all. ICs must self-report their status as caregiver to someone with PF who has used daytime O_2_ for at least eight months. Physicians must have prescribed O_2_ to at least one PF patient.

PF patients in category 4 (no daytime O_2_ at enrollment) must obtain permission from their physicians to wait seven-ten days before starting daytime O_2_. This delay will allow for data collection just prior to them starting O_2_. When these category 4 patients enroll, we will send them a Patient-Doctor Visit Study Packet which includes a letter to both parties reminding them of the terms of the study. The doctor of the patient-participant will sign and fax to the P_3_F team a memorandum agreeing to the terms of the study, including having the participant wait to start daily-use O_2_ for seven-ten days after it is prescribed.

### Study overview

Quantitative data will be collected through questionnaires and GPS and accelerometer devices (see section titled “Quantitative data collection” for more details on these modes of data collection). Quantitative data will be captured once for patients in category 1 and at four time points for patients in category 4 (the longitudinal study): 1) at enrollment; 2) just prior to daily-use O_2_ initiation; 3) one month after starting daytime O_2_; and 4) 9–12 months after starting daytime O_2_.

For patients in the longitudinal arm, beginning at enrollment, we will also collect monthly response data for the University of California San Diego Shortness of Breath Questionnaire (or UCSD). Doing so gives us the opportunity to plot rich trajectories for dyspnea and to detect subtleties that might be missed with a less frequent data collection schedule for this outcome.

Qualitative data will be collected via semi-structured, in-depth telephone interviews and used to clarify and enrich findings from the quantitative data. The interviews will be conducted either once (for patients in category 1) or, for some patients in the longitudinal study, at the four time points described above (see section titled “Qualitative data collection” for a more detailed description of the interviews). The National Jewish Health Institutional Review Board has approved the study protocol (HS-2790), and the study is registered on ClinicalTrials.gov (NCT01961362).

### Quantitative data collection

**Figure 1:**
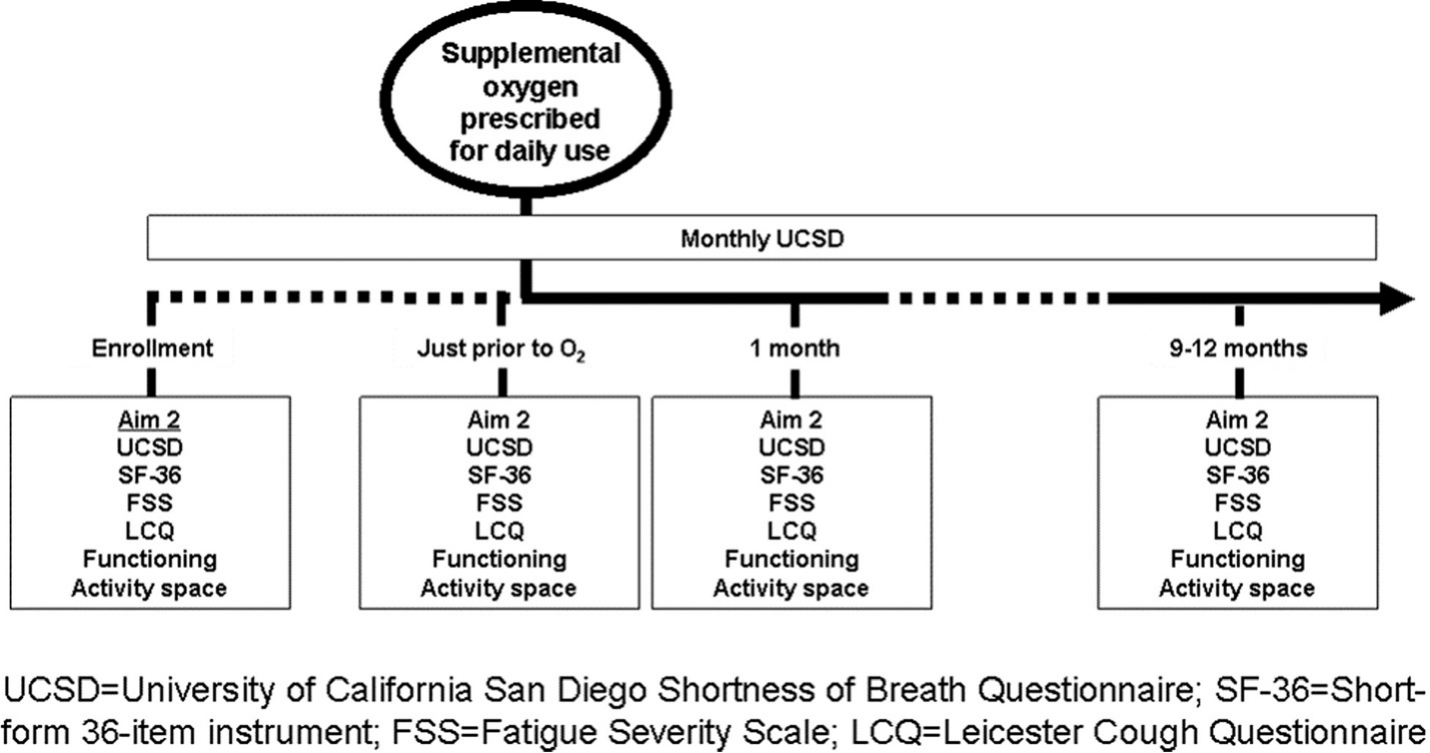
**Longitudinal study design.**

Figure 1 gives an overview of the longitudinal arm of the study. In keeping with the mission and mandates of our program’s funder, the Patient-Centered Outcomes Research Institute (PCORI), our team selected outcome measures by democratic process. During round table discussions, questionnaires were selected from groups of candidate instruments. Questionnaires will be administered and completed online, or, for those who wish, via paper and pencil and returned via pre-paid envelope. Online questionnaires will be completed via REDCap software (http://project-redcap.org/).

#### The UCSD

The UCSD is a 24-item dyspnea questionnaire that asks respondents to rate themselves from 0 (“Not at all”) to 5 (“Maximally or unable to do because of breathlessness”) in two areas: 1) how short of breath they are while performing various activities (21 items); and 2) how much shortness of breath itself, fear of hurting themselves by overexerting, and fear of shortness of breath limit them in their daily lives (3 items). Scores range from 0 to 120, with higher scores indicating greater dyspnea [12]. The UCSD takes 5 minutes to complete.

#### The Short Form 36-Item Instrument (SF-36)

The SF-36 is a generic health-related QOL (HRQL) questionnaire with eight domains which comprise two component summaries (physical and mental). Each domain and component is scored from 0–100, with higher scores connoting greater HRQL [13]. The SF-36 takes 15 minutes to complete.

#### The Fatigue Severity Scale (FSS)

The FSS is a 9-item questionnaire, scored from 9–63, with higher scores indicating more severe fatigue. The FSS takes less than five minutes to complete.

#### The Leicester (pronounced Lester) Cough Questionnaire (LCQ)

The LCQ is a 19-item questionnaire that taps the physical, psychological and social aspects of cough. Scores range from 7–63, with higher scores indicating better cough-related QOL. The LCQ takes five minutes to complete [14].

#### Activity monitor

We are capturing physical activity with an accelerometer—the Actigraph GT3X + Tri-Axis Actigraphy Monitor (http://www.actigraphcorp.com/). It is a small, lightweight (19 grams), plastic device (about the size of a large wristwatch) affixed to an elastic band and comfortably worn around the wrist or waist. The device continuously records data which can be downloaded onto a computer via a USB cable.

#### Activity space

We are using a mobile GPS unit—the iGotU GT-600 GPS data-logger from MobileAction Technologies (http://www.i-gotu.com/)—to capture activity space. These units are small and easily worn or carried around by the participant. They have good reliability and spatial accuracy, even in urban settings [15]. Activity space is often defined as the local areas within which people move or travel in the course of their daily activities and can be used to examine whether people’s mobility changes during the course of medical treatment [16]. Recent research suggests that collecting GPS data on people’s movements is more accurate than travel diaries or semi-structured interviews which ask participants to recall their activities and movements throughout the study period [17, 18]. Data from the GPS loggers will be imported into ArcGIS mapping software and used to create secondary outcome measures which examine the extent of a participant’s activity space and how this changes over time [19–21]. We will ask subjects to keep a trip/travel diary so we can understand where and why trips outside the home were taken.

### Qualitative data

Table 2 describes the interview schedule. To collect a wide range of perceptions of O_2_ we are conducting interviews with 20 PF patients who have used O_2_ during the day for at least eight months. Because O_2_ can potentially affect the entire household of patients who use it (particularly ICs^6^), we are interviewing 20 people who self-identify as ICs of PF patients to get their perspective of how O_2_ impacts them and their patient-loved-ones. To better understand what O_2_ prescribers expect their PF patients to gain (and/or endure) when using O_2_, we will interview 20 O_2_ prescribers. To evaluate how PF patients’ perceptions of O_2_ change over time, we will interview 40 (of the projected 300) subjects enrolled in the longitudinal arm of the study at the four time points mentioned above.

**Table 2:** **Interview schedule**

Category	N	Interview(s)
1. PF patients who have used O_2_ for at least eight months	20	Once
2. ICs of PF patients who have used O_2_ for at least eight months	20	Once
3. Practitioners who have prescribed O_2_ for at least one patient with PF	20	Once
4. PF patients not on daily-use O_2_ at enrollment	40	Four:
		1) At enrollment
		2) Just prior to starting daily-use O_2_
		3) 1 month after starting daily-use O_2_
		4) 9–12 months after starting daily-use O_2_

### Data analysis

#### Qualitative data

The goal of the qualitative analysis is to capture the words participants use to describe their perceptions of O_2_ whether good or bad. They will be asked how oxygen helps or deters them from doing what they need or want to do; how they feel about having to use it; whether there are barriers to its use, and; for patients in the longitudinal arm of the study, how these change over time. Consistent with qualitative methodology, analysis is planned as a continuous process beginning with initial interviews and continuing throughout and beyond the data generation period. After reading and re-reading transcripts to achieve data immersion, data will be coded following a process of initial review, with labeling of data by content, process, or impressions of the investigator. The degree of consensus about particular topics discussed across subjects and over time within the same subject (in the longitudinal arm) will also be noted. After this coding is completed, investigators will organize codes into categories that reflect symbolic domains of meaning, relational patterns within domains, and finally overarching themes. Relationships within domains are usually structured according to “organizing principles”, such as inclusion, symbol, sequence, function, part-whole, or others. Using this analysis, an analytic summary of each interview will be written. In the summaries, the research team will use research participants’ own words and narratives to preserve the tone and emotion of their experiences and increase the theoretical depth of the final description of the effects of O_2_ on PF patients’ lives. Narratives, as a specific kind of speech act in the interview settings, will be indexed in the coding process, and a narrative analysis will ensue. These narratives will be analyzed for substantive and conceptual meaning along with interview discourse. Comparisons across subjects and over time within each subject will add detail and depth to the dimensions of the effects of O_2_.

The software package ATLAS.ti will be used to analyze the qualitative data. As a theory-building qualitative package, ATLAS.ti will be used to code the data, to help the investigators record memos and insights about the data, and to build and test theories.

#### Quantitative data

We will use paired t tests for the primary and secondary endpoints. The standard deviational ellipse (both one and two standard deviations) will be used as measures of activity space [22]. Because we are collecting longitudinal data, in certain exploratory analyses, we will also use longitudinal analytic methods—mixed-effects models will be employed here—to compare the various outcomes across multiple time points while controlling for potentially influential variables. We will conduct certain analyses with the sample stratified on type of pulmonary fibrosis using clinical data acquired from the subjects’ treating physicians. All statistical analyses will be conducted using SAS (SAS, Inc.; Cary, NC).

#### Sample size

For the primary outcome, change in UCSD score from baseline to one month after daily supplemental oxygen use, assuming a conservative correlation of 0.5 between baseline and one-month UCSD scores, a standard deviation of UCSD change scores of 16 [23], and a two-tailed alpha value of 0.05, 83 subjects are needed to have 80% power to detect a 5-point difference in UCSD scores from baseline to one month. We will enroll 300 subjects, conservatively assuming 100 will be prescribed supplemental oxygen during the first two years of the study period.

## Discussion

Our team from the P_3_F has designed a mixed-methods study of O_2_ in PF. The study was, in part, developed by PF patients for PF patients. To ensure an impactful study, our team selected meaningful outcomes that span a diverse spectrum of constructs. For PF patients, whose daily struggles include managing symptoms of cough, fatigue and dyspnea-imposed limitations in physical activity (including activities of daily living), outcomes that assess how patients feel and function seemed most appropriate to our team. Only patients know how they truly feel (physically and emotionally), and using a range of patient-reported outcome measures (PROs) is an ideal way to capture patients’ perceptions.

How best to assess physical functioning among patients with PF is not known. The six-minute walk test (6MWT) is frequently used clinically and in research to assess exercise capacity in PF patients [24]; however, outside the rigorous confines of a therapeutic trial, the 6MWT is fraught with challenges, including poor reproducibility, non-standardized administration and management of O_2_ during the test [25, 26]. Additionally, the 6MWT is an artificial stressor and does not replicate how a patient would normally accomplish the task of walking from point A to point B. Most importantly, the 6MWT may tell nothing about how a PF patient functions around the house or whether they are able or willing to leave their home day-to-day. We expect the accelerometer and GPS data will. Moreover, the 6MWT is unable to incorporate the influence of O_2_ on these parameters. For example, when using O_2_, PF patients may be more willing to leave the home, because they have greater energy and less dyspnea; or, they may not want to deal with the hassles of getting their O_2_ tanks ready or to be seen in public using O_2_. Many patients who need O_2_—not just PF patients—are embarrassed when using O_2_ in public places and perceive they are viewed by others as “sick” or “weak [6, 27, 28]”. These emotions and perceptions may change over time (as patients adapt to their evolving disease state). Our study design will allow us to capture those changes—and resultant behavioral modifications—if they occur.

We recognize that our study has limitations. In choosing the pre-/post- design (as opposed to, say, a randomized trial), we know there is a greater potential for bias. When designing any study, investigators have to make concessions. Our team places great value in the “real world” design and implementation of this study; the downstream effects of this pragmatic approach are that results from this research will be extremely far-reaching—applicable to PF patients nationwide, whereas data from randomized trials often are not.

We have attempted to design several safety nets to ensure data collection occurs just prior to subjects starting daily-use O_2_. Obviously, we will not ask patients who “need” O_2_ urgently (e.g., suffer an acute decline in their PF or are placed on O_2_ to treat another acute process, like pulmonary embolism) to wait before starting daytime O_2_. Research coordinators will know when subjects have appointments with their doctors. They will follow-up with subjects after their appointments to assess whether daily-use O_2_ was recommended/prescribed and implement the seven-ten-day delay for data collection. Having subjects complete the UCSD every month (24 items, 5 minutes to complete) will keep them engaged in the study and make it less likely for data to be missing.

We considered limiting enrollment in the longitudinal study to patients who are not using O_2_ at all (e.g., no nocturnal oxygen). However, what often happens is that patients “ease” into O_2_ use: initially it is prescribed for nighttime use only, and then later—if/when PF progresses—they are prescribed it for daytime use. Excluding subjects already on nocturnal oxygen could significantly hinder our ability to enroll the study. Thus, we decided that patients not using O_2_ during the day (even if they are using it at night) would be eligible. In both qualitative and quantitative analyses, we will examine differences between subjects who, at enrollment, are not on O_2_ at all and those who are using it at night.

## Conclusions

We believe we have achieved a balance between the demands the study imposes on subjects and the benefits of the knowledge the PF field will gain once the study is completed. And despite its limitations, we also believe we have designed an informative study whose successful completion will yield a compilation of data that begins to fill the information vacuum that currently exists around the issue of whether O_2_ benefits patients with PF. These data will propel the field to a new level of understanding of whether/how O_2_ improves how PF patients feel or function and provide the evidence patients and prescribers need to make informed decisions about O_2_. Because of the mixed-methodology we employ in this project, we will also be able to tease out why certain patients do not benefit from O_2_. We will identify the logistical and psychological barriers which prevent people from consistently or effectively using O_2_, and we can use this newfound understanding to begin to devise ways we can all work together—physicians, patients, loved ones, suppliers, manufacturers, etc.—to improve the overall O_2_ experience.
